# Beyond fundamental resonance mode: high-order multi-band ALN PMUT for in vivo photoacoustic imaging

**DOI:** 10.1038/s41378-022-00426-7

**Published:** 2022-11-09

**Authors:** Junxiang Cai, Yiyun Wang, Daohuai Jiang, Songsong Zhang, Yuandong Alex Gu, Liang Lou, Fei Gao, Tao Wu

**Affiliations:** 1 School of Information Science and Technology, ShanghaiTech University, Shanghai, China; 2 Shanghai Institute of Microsystem and Information Technology, Shanghai, China; 3 University of Chinese Academy of Sciences, Beijing, China; 4 School of Microelectronics, Shanghai University, Shanghai, China; 5 Shanghai Industrial μTechnology Research Institute, Shanghai, China; 6 Shanghai Engineering Research Center of Energy Efficient and Custom AI IC, Shanghai, China

**Keywords:** Electrical and electronic engineering, Physics

## Abstract

This paper reports on an aluminum nitride (AlN) piezoelectric micromachined ultrasound transducer (PMUT) array for photoacoustic (PA) imaging, where the high-order resonance modes of the PMUT are utilized to improve imaging resolution. A flexural vibration mode (FVM) PMUT is fabricated and applied in a photoacoustic imaging (PAI) system. Specifically, the microelectromechanical system (MEMS)-based PMUT is suitable for PA endoscopic imaging of blood vessels and bronchi due to its miniature size and high sensitivity. More importantly, AlN is a nontoxic material, which makes it harmless for biomedical applications. In the PAI system, the AlN PMUT array is used to detect PA signals, and the acousto–mechanical response is designed and optimized at the PMUT’s fundamental resonance. In this work, we focus on the high-order resonance performance of the PMUT PAI beyond the fundamental resonance. The acoustic and electrical responses of the PMUT’s high-order resonance modes are characterized and analyzed. The fundamental and three high-order resonance bandwidths are 2.2, 8.8, 18.5, and 48.2 kHz. Compared with the resolution at the fundamental resonance mode, the resolutions at third- and fourth-order resonance modes increase by 38.7% and 76.9% in a phantom experiment. The high-order resonance modes of the AlN PMUT sensor array provide higher central frequency and wider bandwidth for PA signal detection, which increase the resolution of PAI compared to the PMUT working at the fundamental resonance mode.

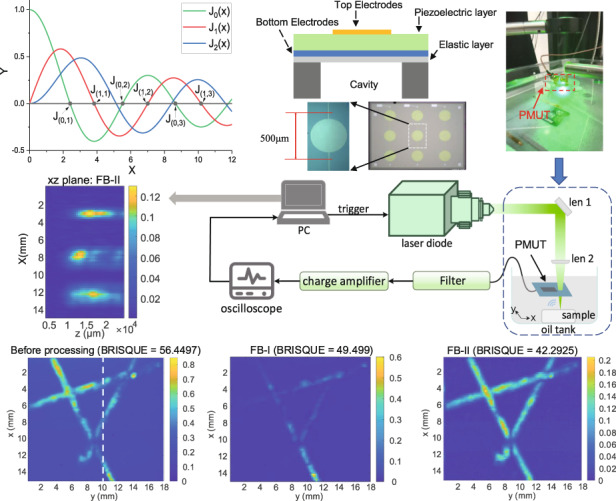

## Introduction

Since the photoacoustic effect was reported by Alexander Bell in 1880s, the phenomenon has been studied in various fields, such as gas sensing^1,2^ and biomedical imaging^3,4^. Photoacoustic (PA) imaging is a physical process that configures laser excitation and ultrasonic reception^4–6^. When a particle is irradiated by a short pulse of laser beam instantaneous thermal expansion and contraction occur due to the particle’s optical absorption. The expansion and contraction generate an acoustic wave that can be received by the ultrasound transducers. As photoacoustic imaging (PAI) has become an emerging and fast-growing imaging technique in the 21st century, researchers have utilized PAI to provide diverse biomedical information and help biological and clinical scientists better understand the biological tissues in certain dimensions. Based on the optical absorption contrast, such as in hemoglobin and melanin, PAI mainly provides functional information, utilizing either endogenous or exogenous contrast agents^7,8^. Therefore, PAI has become an emerging research field and complements ultrasound imaging (USI) by revealing both functional and morphological information^8^.

PAI has been developed with state-of-art ultrasound transducers to achieve high spatial resolution, larger imaging depth and rich optical contrast with smaller, faster, and less expensive systems. PAI is efficient for the early cancer detection of breast, prostate, pancreatic and colorectal cancers^9,10^. Due to the strong attenuation of the excitation laser in human tissues, the image depth is still limited to <5 cm, which makes it hard to diagnose deep information inside the human body, such as blood vessels in viscera. As a result, endoscopy imaging with miniaturization technology is in highly demanded. The use of conventional bulk piezoelectric transducers in endoscopy is limited by their size and fabrication^11^. Fortunately, microelectromechanical system (MEMS) technology can provide a solution to miniaturize transducers for endoscopic PAI applications.. Ultrasound transducers based on MEMS technology can be divided into two types: piezoelectric micromachined ultrasound transducers (PMUTs) and capacitive micromachined ultrasound transducers (CMUTs). CMUTs have the advantages of high sensitivity and tunable wide bandwidth, however, they require high bias voltage^12^ or other voltage reduction technique^13^, which can cause safety risks in biomedical in vivo imaging applications^12^. In contrast, PMUTs are more flexible and safer for in vivo applications because of their passivity^14^.

PMUTs are usually categorized into two types according to the working mode: thickness extension mode (TEM) and flexural vibration mode (FVM). Different types of TEM PMUTs have been fabricated and applied in PAI based on ceramic lead zirconate titanate (PZT)^15–17^, polyvinylidene fluoride (PVDF)^18,19^, single-crystal lead magnesium niobate–lead titanate (PMN-PT), and lithium niobate (LiNbO_3_)^20^. TEM PMUTs based on single-crystal PMN-PT and ceramic PZT have both high frequency and high sensitivity due to the superior piezoelectric constants of PZT and PMN-PT. However, TEM PMUTs have drawbacks of low imaging speed in the application of PAI^12,21^. PMUTs based on PVDF have the advantage of broad bandwidth, but, PVDF’s piezoelectric constants are so low that the transducers have to be made large to ensure enough sensitivity to detect PA signals^22^. PMUTs based on LiNbO_3_, PMN-PT, or PZT films can be fabricated into smaller sizes, but their process is incompatible with modern complementary metal-oxide semiconductor (CMOS) technology. Compared to the TEM PMUTs, FVM PMUTs have relatively lower acoustic impedance and are easier to integrate multiple frequency bands. Moreover, an FVM PMUT is easier to manufacture and form arrays for higher sensitivity, larger bandwidth and more functionality. In the past decades, ZnO, PZT, and AlN thin films have been widely used to fabricate FVM PMUT^23–26^. Compared with ZnO and PZT, AlN has better chemical and thermal stability as well as biosafety, and the AlN PMUT device fabrication is also compatible with modern CMOS fabrication processes^27–29^, which makes the CMOS-MEMS monolithic transducer chips possible. In addition, the sensing sensitivity is proportional to *e*_3.1.f_/*ϵ*_33.f_ (e_3.1.*f*_:piezoelectric constant, ϵ_33.*f*_:dielectric constant.) for FVM piezoelectric MEMS transducers, therefore, AlN thin film is preferred for higher sensitivity compared to PZT due to their small dielectric constant^30^. Recently, AlN thin films-based FVM PMUTs have been implemented in ultrasonic imaging^31^, finger-print identification^32^, and PA applications^33^.

In PAI, both lateral and axial resolutions can affect imaging quality. The lateral resolution is determined by the overlap of optical excitation and ultrasonic detection^3^. The axial resolution originates from the PA signal’s full width of half maximum (FWHM) and it is proportional to the acoustic detector’s bandwidth. Typically, a PA signal has a short pulse profile with a wide bandwidth. It is necessarily required to use an acoustic sensor with a wide bandwidth to acquire PA signal with high fidelity. Therefore, increasing the bandwidth of the transducer is highly essential to improve PAI resolution.

To address the challenges of smaller bandwidth in FVM PMUTs for PAI, currently, there are two ways to expand the bandwidth of the transducer: combining multiple PMUTs with different dimensions (different resonance frequencies) into one array^34,35^ and designing rectangle structures PMUT with multiple resonance modes^36–38^. In this paper, circular FVM AlN PMUT’s higher order resonance modes are used to expand the bandwidth of the transducer in PAI, beyond the fundamental resonance mode. We use a lumped element model (LEM) to analyze the modes of AlN PMUT coupling. We discuss the performance of PMUT in the phantom and in vivo experiments under several frequency bands. The imaging performance is analyzed in terms of image quality, lateral resolution, and axial resolution. The comparisons in the axial resolution among different frequency bands are discussed. It is shown that PMUT at a fundamental resonance mode has difficulty performing high-resolution imaging, while a sensor with high-order resonances displays much higher resolution with a better 3D imaging quality.

## Materials and methods

An acoustic transducer plays a crucial role in the endoscopic PAI systems. As the acoustic sensor, the PMUT receives the PA signals excited by the pulsed laser. This kind of microsensor based on MEMS technology can be implanted in a living body for healthcare monitoring. In this section, the structural design, circuit model, and fabrication process of an FVM PMUT based on AlN thin film are presented and analyzed.

### PMUT structural design and modeling

**Fig. 1:**
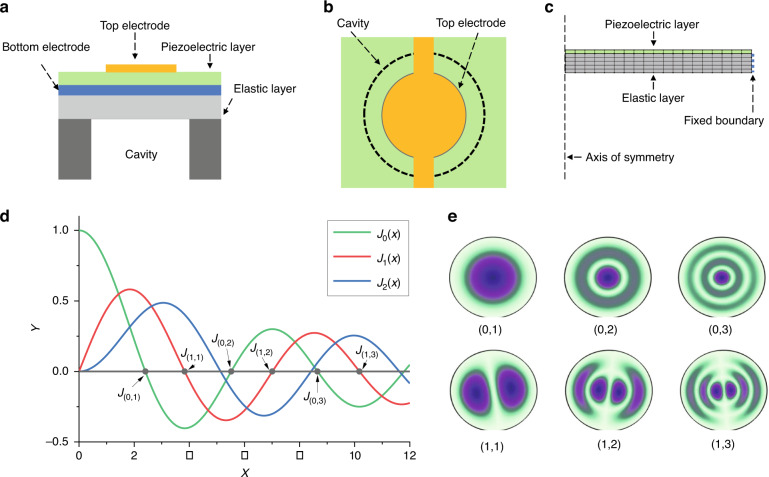
Mode shape analysis of PMUT. **a** Cross-sectional view and **b** top view of FVM PMUT; **c** FEM axisymmetric 2D model; **d** Curves of Bessel function of the first kind; **e** common vibration modes of a circular membrane with a fixed rim boundary.

The designed PMUT works in a flexural vibration mode. As it is shown in Fig. 1a, the PMUT element, which is essentially a bilayer diaphragm, contains a piezoelectric layer is sandwiched between a the top electrode and bottom electroelectrodes and an elastic layer. The cavity shape defines the PMUT’s effective fixed boundary. The flexural vibration is primarily actuated by the d_31_ mode excitation of the piezoelectric diaphragm. Figure 1b shows the top view of the structure with a circular membrane design. The effective area of the top electrode is ~70% of the cavity^39,40^, which is an optimized design for the fundamental vibration mode.

The vibration modes of a circular PMUT with a fixed membrane boundary can be derived from a specific form of Helmholtz equation^41^. Assuming a circular membrane is fixed at a radius of *r* = a, the equation can be expressed as:

1 $$\frac{{\partial ^2\Psi }}{{\partial r^2}} + \frac{1}{r}\frac{{\partial \Psi }}{{\partial r}} + \frac{1}{{r^2}}\frac{{\partial ^2\Psi }}{{\partial \theta ^2}} + k^2\Psi = 0$$

where Ψ is the function of position, and wave number *k* = ω/c. *c* is the speed of sound. Ψ(*r*, *θ*) is the product of two independent spatial function terms,

2 $$\Psi = R\left( r \right)\Theta \left( \theta \right)$$

where *R*(a) = 0, and Θ(*θ*) is a smooth and continuous function of *θ*. After separating the variables and considering the specific physical facts, the two functions have the following solution forms:

3 $$\Theta \left( \theta \right) = \cos \left( {{{{\mathrm{m}}}}\theta + \gamma _{{{\mathrm{m}}}}} \right)$$

4 $${{R}}\left( {{r}} \right) = {{{\mathrm{AJ}}}}_{{{\mathrm{m}}}}\left( {{{{\mathrm{kr}}}}} \right)$$

In the solution of Θ, *m* is an integer because Θ must be smooth and single-valued, and the γ_m_ values are determined by the initial conditions of the membrane. Solution R conforms the solution of the Bessel equation, and *J*_m_ is the Bessel function of the first kind. Figure 1d shows the first three curves of the Bessel function of the first kind. In Eq. (4), the boundary condition requires *J*_m_(ka) = 0, so each of the zero point of the Bessel function of the first kind corresponds to a normal vibration mode^41^. Assumes k_mn_ a is all the value that makes *J*_m_ = 0, then the solutions of vibration amplitude are described as:^41^

5 $${{y}}_{{{{\mathrm{mn}}}}}\left( {{{r}},\theta ,{{t}}} \right) = {{A}}_{{{{\mathrm{mn}}}}}{{J}}_{{m}}\left( {{{k}}_{{{{\mathrm{mn}}}}}{{r}}} \right)\cos \left( {{{m}}\theta + {\upgamma}_{{{{\mathrm{mn}}}}}} \right){{{\mathrm{e}}}}^{{{{\mathrm{j}}}}\omega _{{{{\mathrm{mn}}}}}{{t}}}$$

Figure 1c shows the vibration mode shapes analysis setup of a circular membrane with a fixed rim boundary from an axisymmetric 2D model based on the COMSOL finite element method (FEM). The top view of several common vibration modes from the FEM analysis is illustrated in Fig. 1e. These fixed boundaries, at which the displacement amplitude is always zero as nodal lines, are described by Eq. (5) and the Bessel curves in Fig. 1d. For example, fundamental mode (0,1) in Fig. 1e has only one circular nodal line (physical boundary) along the radius. As a result, the resonant vibration can be divided by mode (*m*, *n*), where m is a natural number (0,1,2…) and n is the counting number (1,2,3…). “*m*” determines the number of radial nodal lines and “*n*” determines the number of nodal circles^41^.

Lumped element modeling (LEM) is a powerful tool for both designing and analyzing multiphysics systems. LEM can simplify the description of spatially distributed physical systems by using a set of lumped elements under certain assumptions, Several LEM approaches have been studied to analyze the responses of PMUTs in photoacoustic environment^42–44^. A simplified version LEM has been built for the FVM PMUT to analyze the transformation of energy in different domains.

**Fig. 2:**
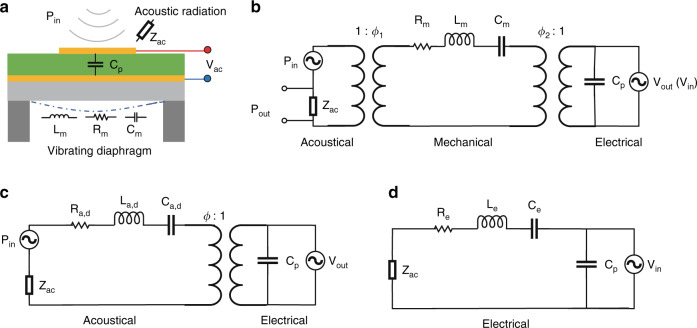
Circuit models of PMUT. **a** Illustration of the PMUT structure with lumped elements; **b** Lumped element modeling (LEM) circuit model of a PMUT in three domains. **c** Simplified LEM circuit model in two domains; **d** BVD electrical model of FVM PMUT.

The illustration of the FVM PMUT structure with the lumped elements is shown in Fig. 2a, which includes an electric interface, the acoustic radiation from the medium, and the vibrating diaphragm of mechanical vibrating film. Figure 2b illustrates the LEM circuit of the FVM PMUT, which contains the acoustical, mechanical, and electrical domains. In the equivalent circuit model, the acoustic domain is coupled to the mechanical domain through a transformer of a coefficient Φ_1_, while the mechanical domain and electrical domain are coupled by another transformer with a coefficient Φ_2_. The circuit model indicates that the PMUT can work as both a sensor and an actuator.

In the LEM circuit model shown in Fig. 2b, the lumped element *Z*_*ac*_, also indicated in Fig. 2a, represents the acoustic radiation impedance of the membrane; C_d_, R_d_, and L_d_ are the equivalent circuit elements for the mechanical compliance, mechanical damping, and effective mass of the membrane; and C_p_ is the measured electrical capacitance of the sandwiched piezoelectric layer^45^.

In PAI applications, PMUTs work as only acoustic sensors to receive photoacoustic signals. With the known acoustical-mechanical coupling coefficient Φ_1_, the LEM circuit model can be simplified as in Fig. 2c. According to the new FEM elements, we can derive the critical parameters of FVM PMUT. The resonant frequency of the PMUT membrane f_0_:

6 $${{{\mathrm{f}}}}_0 = \frac{1}{{2{\uppi}}}\sqrt {\frac{1}{{{{{\mathrm{L}}}}_{{{{\mathrm{a}}}},{{{\mathrm{d}}}}}{{{\mathrm{C}}}}_{{{{\mathrm{a}}}},{{{\mathrm{d}}}}}}}}$$

And the quality factor *Q* can be expressed as:

7 $${{{\mathrm{Q}}}} = \frac{1}{{{{{\mathrm{R}}}}_{{{{\mathrm{a}}}},{{{\mathrm{d}}}}} + {{{\mathrm{Re}}}}\left( {{{{\mathrm{Z}}}}_{{{{\mathrm{ac}}}}}} \right)}}\sqrt {\frac{{{{{\mathrm{L}}}}_{{{{\mathrm{a}}}},{{{\mathrm{d}}}}}}}{{{{{\mathrm{C}}}}_{{{{\mathrm{a}}}},{{{\mathrm{d}}}}}}}}$$

By knowing f_0_ and *Q*, we can easily derive the bandwidth of the FVM PMUT:

8 $$\Delta {{{\mathrm{f}}}} = \frac{{{{{\mathrm{f}}}}_0}}{{{{\mathrm{Q}}}}} = \frac{1}{{2{\uppi}}}\frac{{{{{\mathrm{R}}}}_{{{{\mathrm{a}}}},{{{\mathrm{d}}}}} + {{{\mathrm{Re}}}}\left( {{{{\mathrm{Z}}}}_{{{{\mathrm{ac}}}}}} \right)}}{{{{{\mathrm{L}}}}_{{{{\mathrm{a}}}},{{{\mathrm{d}}}}}}}$$

Each resonance of the PMUT has a corresponding L_d_, R_d_, C_d_. At the fundamental resonance, the sensor shows a large displacement magnitude, which reflects its high sensitivity. The equivalent small R_d_ corresponds to a high-quality factor (*Q*), which indicates a narrow bandwidth. In addition, other high-order resonances have a high equivalent R_d_, which leads to a lower *Q* that leads to a broad frequency band and less oscillation. The series resonance of L_d_ and C_d_ represents the resonance of the PMUT.

Typically, the PMUT’s membrane behaves like a linear resonator for small displacement. If we transfer lumped elements of the acoustical domain into the electrical domain illustrated in Fig. 2c, we can obtain a Butterworth–Van–Dyke (BVD) electrical model of an FVM PMUT as shown in Fig. 2d. Similarly, the C_p_ is also the static capacitance of the sandwiched piezoelectric element as mentioned in models (a) and (b). The resistor R_e_ models the overall losses of the structure, while the inductor L_e_ models the effective mass and the capacitor C_e_ models the equivalent compliance counting the cross-energy domain couplings. Impedance *Z*_*ac*_ represents the interface of the acoustic domain.

### PMUT device and system

**Fig. 3:**
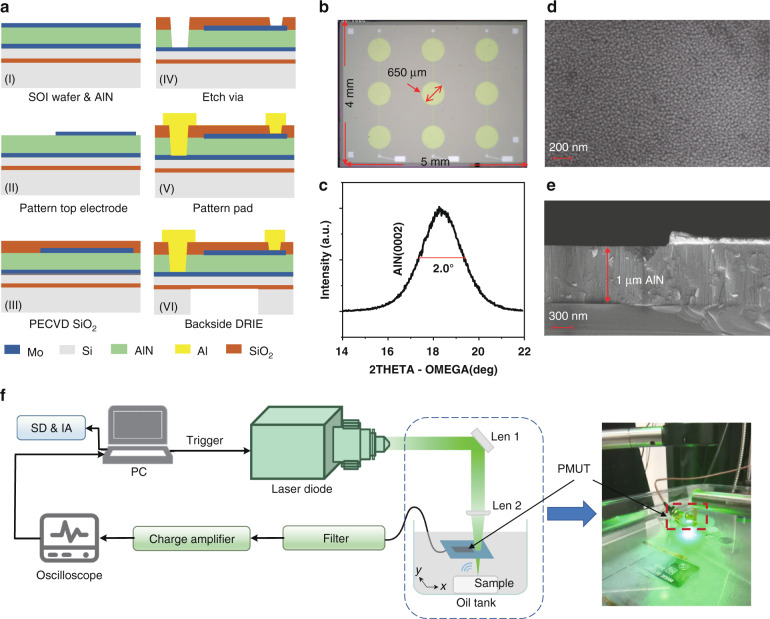
Fabrication of PMUT array and schematic design for photoacoustic imaging experiment. **a** Process flow. **b** Optical image of PMUT. **c** 2θ-ω scan XRD rocking curve of the AlN thin film. **d** Surface of AlN thin film captured by SEM. **e** Cross-sectional view of deposited AlN thin film captured by SEM. **f** PAI experiment setup. Len 1: mirror; len 2: plano-convex lens; PC personal computer, SD & IA signal decomposition and image analysis.

Figure 3 shows the circular PMUT fabrication and PA characterization. As illustrated in Fig. 3a, the Mo/AlN/Mo film stacks are sputtered on a silicon-on-insulator (SOI) wafer. AlN film, deposited at 300 °C by DC-pulse reactive magnetron sputtering, achieves average stress within +/− 50 MPa and a stress range within 200 MPa based on our previous work^46,47^. The top patterned Mo electrode is typically designed at a size of 65–70% of the cavity diameter in order to effectively collect the charge of AlN piezoelectric layer. The AlN layer is ~1 μm, while the top and bottom Mo layers are ~200 nm. The SOI box layer is 1 μm, and SOI Si device layer is 5 μm. The top PECVD silicon dioxide (SiO_2_) layer serves to passivate the PMUT cell and prevent Mo oxidation. Through two-step etching (III–IV), 1 μm routing and pad metal Aluminum (Al) connects the bottom and the top electrodes to the surface through opened vias (V). Finally, a backside DRIE process (VI) creates cavities, which define the effective flexural membrane diameter and boundary of each PMUT cell. The optical picture of the PMUT array is shown in Fig. 3b. The PMUT array contains three individual channels, and each channel contains three same cells with top electrodes connected together. The radius of each cell is ~500 μm. Figure 3c is the 2*θ*−*ω* scan XRD spectrum of the AlN thin film, where the rocking curve FWHM of the AlN film is ~2°, indicating good film deposition quality. Figure 3d, e shows scanning electron microscopic (SEM) images of surface morphology and a cross-sectional view of AlN thin films. The SEM images indicate that the AlN piezoelectric layer has a smooth surface and vertical crystallographic orientation, which also confirms the excellent quality of the AlN film from XRD rocking curve analysis.

### Photoacoustic imaging experiment setup

The experimental setup of the photoacoustic imaging is shown in Fig. 3e. The fabricated PMUT array devices are mounted on a customized PCB through wire bonding and positioned inside an oil tank in close to the imaging samplings. A 532-nm pulsed laser (DPS-532-A, CNI Laser) generates laser illumination, traveling through the tiny hole on the PMUT’s PCB board. All components are fixed except that the sample is translated by an *x–y* stage positioner in order to form PA imaging. We keep the laser energy constant during the following three experiments. The excited sample absorbs the pulsed laser energy and emits PA waves due to thermoelastic expansion. The PA wave is then received by the PMUT array connected to the PCB. After that, the PA signal passes through an analog high-pass filter to reduce the low-frequency noise under 20 kHz and is then amplified through a charge amplifier. The magnification of the charge amplifier is ~1V/10pC. The preprocessed PA signal is acquired and averaged eight times by an oscilloscope (DPO5204B, Tektronix Inc.), and transferred to the personal computer (PC). The sample is scanned along the *x* and *y axes* with a 0.2-mm scanning step remotely programmed and controlled through the PC.

## Results

### PMUT device characterization

The PMUT array was characterized in terms of frequency-dependent electrical impedance, mechanical vibration amplitude, and mode mapping.

**Fig. 4:**
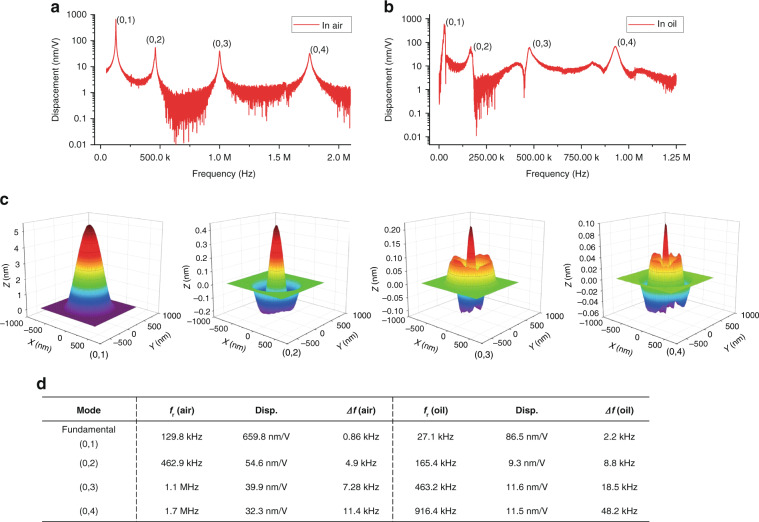
The LDV measurement results of the PMUT. **a** Vibration spectrum in air and **b** in mineral oil; **c** 3D vibration mode measured by LDV; **d** a list of measurement key parameters.

The mechanical vibrations of the fabricated PMUT elements were measured separately in air and in mineral oil by a laser Doppler velocimetry (LDV, MSA-600 Micro System Analyzer, Polytec, Germany). In the air measurement, The PMUT array was electrically driven at 1 V_pp_ with a 20 kHz–2 MHz sweeping frequency. The laser beam hits the center cell of the PMUT array, which is indicated in Fig. 3b. Consequently, the center cell’s vibration was detected. Figure 4a shows the frequency response of PMUT measured in air by LDV. Four resonance peaks are observed in Fig. 4a, indicating the resonance frequencies from low to high corresponding to modes (0,1), (0,2), (0,3), and (0,4), respectively. The relevant resonant frequency displacement and bandwidth data are listed in Fig. 4d. In the mineral oil measurement, the PMUT array was completely submerged in oil. The center cell of the PMUT was electrically driven at the same as in the air measurement. Figure 4b shows the frequency response of the PMUT measured in mineral oil by LDV. Figure 4c shows 3D vibration mode images corresponding to four vibration resonance responses in Fig. 4a. The measured results of the 3D mode shape are in line with the results of the formula derivation and simulation in Fig. 1. The result also shows only (0, *n*) mode can be effectively cited in this kind of sandwich structure design.

The LDV measurements show that as the resonance frequency increases, the bandwidth at the resonance also increases. As shown in the measured data of the air group, the bandwidth of mode (0,1) is 0.86 kHz (calculated by Eq. (3)). Similarly, the bandwidths of (0,2), (0,3), and (0,4) modes are 4.9, 7.28, and 11.4 kHz. The frequency response of the PMUT measured in mineral oil has the same characteristics, in which the bandwidths of four resonances are 2.2, 8.8, 18.5, and 48.2 kHz, respectively. It can be seen that the resonant frequency of the PMUT working in oil is lower than that in air for each corresponding mode, however, the trend of bandwidth is different. The LDV measurement data indicate significant drops in resonance frequency and *Q* factor when the PMUT is working in a liquid environment compared with the same device working in the air. The decrease in frequency is not conducive to the lateral resolution, but the increased bandwidth is in favor of the axial resolution improvement. Therefore, a high-order resonance of PMUT array could be utilized to improve the PAI signals when compared to pure fundamental mode signal characterization.

**Fig. 5:**
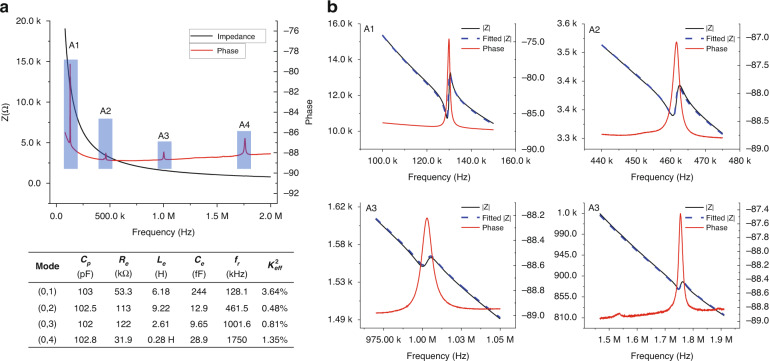
Electrical characteristics of PMUT. **a** Impedance and LEM parameters of PMUT's vibration modes (0,1), (0,2), (0,3) and (0,4) ; **b** BVD fit of the four resonances.

The electrical impedance of the PMUTs was measured in air with an impedance analyzer (E4990A Impedance Analyzer, Keysight, USA). Figure 5a shows the impedance and phase of the PMUT from 20 kHz to 2 MHz measured in air. The black line shows the trend of the absolute value of impedance with frequency, and the red line shows the trend of phase with frequency. As shown in Fig. 5a, the impedance has resonance and anti-resonance at approximately 129 kHz, 462 kHz, 1.1 MHz, and 1.7 MHz, respectively, which corresponds to the LDV measurement result shown in Fig. 4a. The four panels of Fig. 5b are enlarged representations at each resonant frequency. This typical impedance curve with resonant frequency and anti-resonant frequency can be fitted by the BVD model according to the equivalent circuit model of Fig. 2d. The blue dotted lines of Fig. 5b indicate the BVD fitted curve, and the corresponding fitted parameters are recorded as well. For the case of electric input only, the acoustic radiation impedance Z_ac_ in model Fig. 2d can be ignored. As a result, the fitted C_0_ equals capacitance of the sandwiched piezoelectric element C_p_, and L_e_, R_e_, C_e_ are the corresponding equivalent effective mass, mechanical damping, and effective mechanical compliance of the membrane transferred to the electric domain. In the impedance measurement of the air group, the resonance peak of the impedance gradually weakens with the increase of the resonance order.

As the measured resonant frequency f_r_ and the anti-resonant frequency f_a_ are known by impedance measurement, the effective electromechanical coupling coefficient ${{{\mathrm{k}}}}_{{{{\mathrm{eff}}}}}^2$ of the PMUT can be calculated by:^48^

9 $${{{\mathrm{k}}}}_{{{{\mathrm{eff}}}}}^2 = 1 - \left( {\frac{{{{{\mathrm{f}}}}_{{{\mathrm{r}}}}}}{{{{{\mathrm{f}}}}_{{{\mathrm{a}}}}}}} \right)^2$$

The calculated ${{{\mathrm{k}}}}_{{{{\mathrm{eff}}}}}^2$ of the fundamental resonance is 3.64%, and the values of other high-order resonances are 0.48%, 0.81%, and 1.35%. Compared with the fundamental resonance modes, the ${{{\mathrm{k}}}}_{{{{\mathrm{eff}}}}}^2$ of the high-order resonance modes decreased significantly, which could attribute to the reduction in mechanical compliance of the high resonance modes^49^. However, the ${{{\mathrm{k}}}}_{{{{\mathrm{eff}}}}}^2$ of mode (0,4) is higher than modes (0,2) and (0,3), because the 70% electrode is more conducive to capturing the induced electric charge of this mode shape^49^.

### Photoacoustic signal

The PA signals captured from the oscilloscope go through post-processing. According to the PMUT device characteristics in the frequency domain, we extract the photoacoustic signals of four frequency bands around the fundamental and other resonance frequencies. The selected frequency bands are 25–48.9 kHz (FB-I), 373–780 kHz (FB-II), 746 kHz–1.56 MHz (FB-III), and 1.5–3.1 MHz (FB-IV). The envelope of PA signals is extracted in the selected frequency bands. After that, we use the maximum amplitudes of the envelope signals for *xy*-plane imaging by placing them according to the coordinates. We obtain images of the *xz*-planes and *yz-*plane in cross-sectional view with a group of the envelopes on the *x* or *y* axis, sorting them with coordinates.

**Fig. 6:**
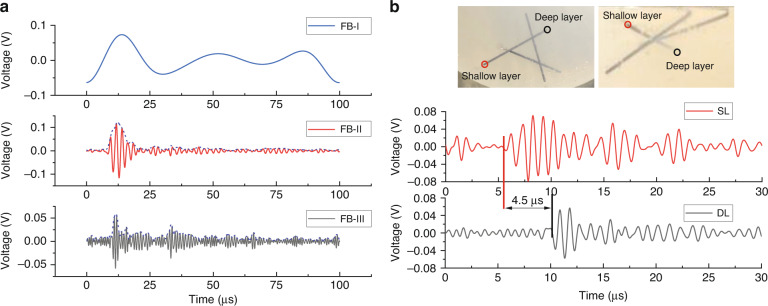
PA signals with different bands and PA signals in different depths. **a** PA signal at DL in three frequency bands through wavelet decomposition. **b** Comparison of PA signal at shallow layer and deep layer in FB-III.

We analyze the performance of different frequency bands’ results in terms of the signal waveform. As illustrated in the device characterization section, the received photoacoustic signal by the PMUT array has the most signal components at its fundamental resonance frequency band (FB-I), and fewer components at other resonant frequency bands. As shown in Fig. 6a, the maximum amplitude of the processed PA signal descends with the signal’s ascending frequency band. At the FB-I (fundamental resonance), the detected PA signal’s amplitude is approximately 0.1 V, and according to the magnification of the amplifier, it can be known that the PMUT generates a 1pC charge. The signal amplitude of the FB-II (mode (0,3)) is also ~0.1 V (should be smaller than FB-I), which means the PA’s signal is mainly concentrated in this frequency band. The amplitude of FB-III (mode (0,4)) is ~0.05 V, which is smaller than the first two, however, two prominent peaks could reveal the sample’s depth information. Typically, the sensor’s imaging performance of the axial resolution is related to the enveloped PA signal’s full width at half maximum (FWHM) in the point-to-point scanning. The FWHMs of signals in FB-I, FB-II, and FB-III are 9.61, 5.89, and 2.22 μs, descending with the ascending frequency band. In other words, the corresponding spatial resolutions are 14.4, 8.8, and 3.33 mm. This shows that the resolutions at the second and third-order resonance modes increase by 38.7% and 76.9%, compared with the resolution at the fundamental resonance mode. The results also indicate that imaging with a high-frequency band should have a better axial resolution, which will be further discussed in the following experiments. If we design and fabricate the sensor with a higher frequency, the axial resolution may be further improved. Figure 6b demonstrates the PMUT array’s ability to detect and discriminate the signal’s depth information. We present two photoacoustic signals in FB-III that are excited from one pencil lead at different depths. Compared with the signal from the shallow layer, the PA signal from the deep layer shows a latency of 4.5 μs, which indicates an ~7 mm depth difference according to the acoustic velocity of 1500 m/s in mineral oil.

**Fig. 7:**
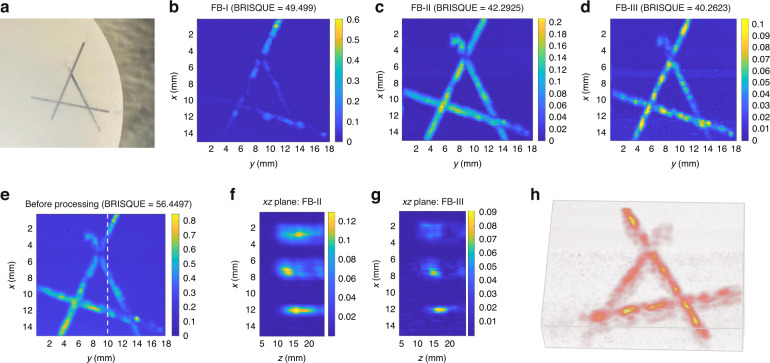
Photoacoustic imaging experiment on the sample. **a** Letter “A” sample: three inclined pencil leads are inserted into the agar phantom. The pencil leads’ diameter is 0.5 mm. **b**–**d** PA images of *xy*-plane cross section at three frequency bands. **e** PA imaging of *xy*-plane cross section before processing. **f**, **g** PA images of *xz*-plane cross section at two resonance frequency bands (FB-II, FB-III). **h** Three-dimensional PA image in FB-III.

The phantom shown in Fig. 7a is scanned by the step motor in both the *x* and *y* axes. Figure 7b–d shows the reconstructed PA images in the *xy*-plane. It is shown that the image through FB-I can hardly identify the letter “A”. The PA imaging through FB-II shows clear pencil leads’ outlines though their depth information is obscure. The pencil leads’ diameter in imaging at FB-III are the closest to the actual diameter, which implies it has the best lateral resolution among the three frequency bands’ results. In addition, the image of FB-III provides more depth information than that in the images of other frequency bands. Here, we choose blind/referenceless image spatial quality evaluator (BRISQUE)^30^ to quantify the image quality. The smaller BRISQUE score indicates better image quality. The BRISQUE values of four PA images: before processing (Fig. 7 e), in FB-I (Fig. 7b), FB-II (Fig. 7c), and FB-III (Fig. 7d), are 56.45, 49.50, 42.29, and 40.26, respectively. Thus, PA imaging in FB-III has the best image quality with BRISQUE. This is mainly due to the lower oscillation at this high-frequency band, which well fits the analysis in the device characterization. Then, the imaging performance in the cross-sectional plane is analyzed. Due to the high signal oscillation at the fundamental resonance mode shown in Fig. 6a, the cross-sectional imaging of FB-I cannot be reconstructed. As shown in Fig. 7e, we select a *xz*-plane cross section at *y* = 10 mm to demonstrate the cross sections of pencil leads at different locations and depths. The imaging result of FB-II in Fig. 7f presents a clear view of leads’ positions. The FB-III’s PA image in Fig. 7g reveals leads’ widths that are closer to the actual widths with relatively weak signal amplitudes. As the signal component’s frequency increases, the penetration depth of the corresponding filtered PA signal decreases. This will lead to relatively weaker signal amplitudes. Thus, shown in FB-III’s image in Fig. 7g, the pencil lead around the *x* = 3 mm depth is almost invisible. Finally, we reconstructed the 3D PA image in Fig. 7h, showing the overall picture of the phantom. Our PAI analysis has demonstrated that the high resonance modes can be utilized to achieve better imaging quality with the help of larger bandwidth.

**Fig. 8:**
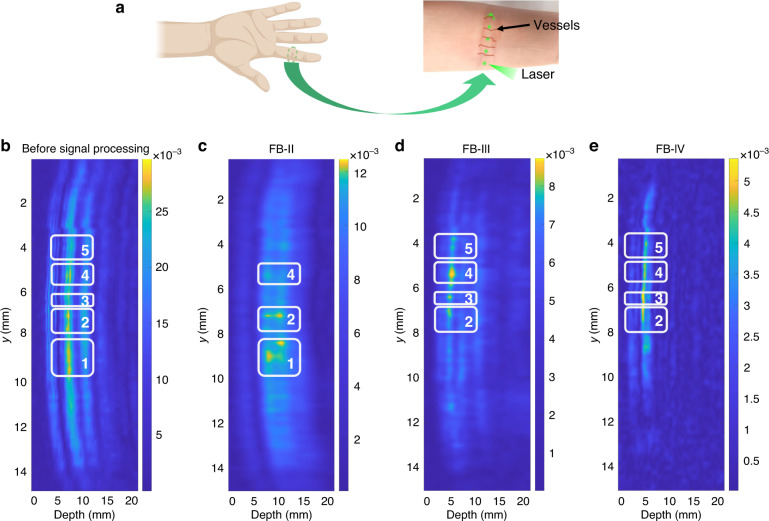
PAI in vivo characterization of a human hand. **a** Schematic diagram (left) and zoom in (right) of the blood vessels in the human index finger; **b**–**e** Cross-sectional images before and after signal processing with different frequency band filters.

To evaluate the PMUT sensor’s performance in clinical scenarios, we conduct the imaging experiment on the human finger joint (Fig. 8a). Human’s finger joint contains multiple vascular plexus and capillary loops. The laser points are scanned through several possible locations to detect the PA signals from the vascular plexus and capillary loops underneath, as indicated by the green laser dots and red vessel lines. Here, we present a cross-sectional PA imaging with various frequency bands. Amplitude mode lines (A lines) of photoacoustic signals are collected to represent the *z* direction, i.e., the depth of the finger joint. As before, we first extract signal components in three frequency bands from the noisy original PA signal by wavelet decomposition. The following illustrates the imaging results before signal post-processing, and in three frequency bands. The image without wavelet decomposition in Fig. 8b shows the least vessel information. Five regions that may include vessels are boxed. Vessels in boxes 1 and 2 become clearer in the FB-II’s image (Fig. 8c). We may observe obscure but deep vascular sections in this relatively low-frequency band. To obtain clear views of the vessels, higher frequency bands are used. Comparing the vascular section in box 4 of the four bands, the cross section of capillaries is visible in the high-frequency band FB-III and FB-IV. As shown in Fig. 8d, e, vascular sections in boxes 3–5 are clearer in images under FB-III and FB-IV, while the sections in boxes 1 and 2 are sharper in the FB-I’s imaging. This indicates that image of the FB-II image may visualize better vessel sections within certain depths, while the images under FB-III and FB-IV may display the vessels in the epidermis layer of skin, the diameter of which is closer to that of the actual ones. These results demonstrate that the current photoacoustic imaging system based on PMUT technology can discriminate vessels at different layers.

## Discussion and conclusion

For clinical imaging scenarios, we require both high resolution and imaging depth for various tissue imaging, which would demand transducer with both relatively low-frequency bands and relatively high-frequency bands with large bandwidth, to reveal comprehensive tissue information. Our PAI analysis indicates that multiple bands of single-type PMUT design can be utilized for different biomedical information acquisition.

In this work, an FVM PMUT array based on an AlN piezoelectric thin film has been designed and fabricated, and the multiple resonance modes of the FVM PMUT are modeled, analyzed, and experimentally verified for PAI applications. The characteristics of the fabricated PMUT’s multi-band resonance have been evaluated with laser Doppler vibration measurements, electrical impedance measurement, and PA signal sensing. The voltage-to-displacement transduction gains of multiple resonance modes in air and water have been measured and compared. The electrical impedance of multiple resonance modes in the air have been fitted by a BVD model and the effective electromechanical coupling ${{{\mathrm{k}}}}_{{{{\mathrm{eff}}}}}^2$ has been calculated. Compared with fundamental resonance, the voltage-to-displacement transduction gain and the effective electromechanical coupling coefficient are smaller, but the corresponding center frequency and bandwidth are increased, which improves the imaging resolution of PAI. The results of PAI on cross-pencil-lead phantom “A” indicates that the lateral resolution of imaging is gradually improved in an increase of the resonance order. The extract FWHM signals indicate that the resolutions at third and fourth-order resonance modes can be improved by ~38.7% and 76.9% compared to the fundamental resonance mode in our PMUT array PAI experiment. It should also be mentioned that results with high-order resonance mode show less sensitivity. Though the trade-off between sensitivity and bandwidth is inevitable, there are ways to compensate the sensitivity loss, such as an increase in laser energy and amplification of the signal processing stage.

We also conducted in vivo photoacoustic imaging experiment on a human finger joint with high-order resonance modes. The PAI result shows the system’s ability to discriminate vessel sections at different layers, which is very close to the actual anatomical positions. The experimental results have demonstrated the great potential of applying this high-order multi-band AlN PMUT array for PAI to provide high lateral and axial resolution of 3D images. In the future, we aim to make and develop a complete endoscope with optical fiber, scanner, and PMUT arrays fully integrated. In addition, PMUTs with special electrodes or structural designs for higher modes may exhibit better bandwidth performance and could be further explored for PAI applications.
